# Factors affecting fistula failure in patients on chronic hemodialysis: a population–based case–control study

**DOI:** 10.1186/s12882-018-1010-6

**Published:** 2018-08-22

**Authors:** Cheng-Chieh Yen, Ching-Fang Tsai, Yueh-Yun Luo, Hsin-Yi Yang, Mei-Yin Liu, Peir-Haur Hung, Yueh-Han Hsu

**Affiliations:** 1 Division of Nephrology, Department of Internal Medicine, Ditmansion Medical Foundation Chia–Yi Christian Hospital, No.539, Zhongxiao Rd.60002, East Dist, Chia–Yi City, Taiwan; 2 Department of Medical Research, Ditmanson Medical Foundation Chia–Yi Christian Hospital, Chia–Yi City, Taiwan; 3 Department of Nursing, Ditmanson Medical Foundation Chia–Yi Christian Hospital, Chia–Yi City, Taiwan; 4 Health Center of Houbi District, Tainan City, Taiwan; 5 Department of Applied Life Science and Health, Chia–Nan University of Pharmacy and Science, Tainan City, Taiwan; 6 Department of Medical Research, China Medical University Hospital, China Medical University, Taichung City, Taiwan; 7 Department of Nursing, Min–Hwei College of Health Care Management, Tainan City, Taiwan

**Keywords:** Diabetes mellitus, Dialysis frequency, Dialysis provider level, Fistula care, Hemodialysis, Late fistula failure, Quality improvement, Taiwan

## Abstract

**Background:**

With advancement of hemodialysis (HD) technique, late fistula failure (LFF) remains a problem significantly affecting life quality of patients. We attempt to identify factors affecting LFF in patients on chronic HD in Taiwan from the National Health Insurance Research Database.

**Methods:**

This case–control study enrolled patients over 18 years old and who received regular HD for more than 3 months. LFF was defined as the first fistula failure episode beyond 3 months of chronic HD. We analyzed characteristics, comorbidities and medicine and investigated the association factors of LFF by logistic regression model. A trend test was conducted for risk in different provider levels. Sensitivity tests were conducted to test consistency.

**Results:**

Of 1558 patients recruited, 772 (49.6%) were identified as LFF cases and 786 were identified as controls. The data showed that patients with diabetes mellitus (DM) had 42% increased rate of LFF. Patients receiving more than 10 HD sessions per month had a 90% increased rate of LFF; patients receiving chronic HD in private clinics had a 49% reduction rate of LFF. There were no significant differences in age, dialysis frequency, and comorbidities among different provider levels. There was a significant trend of risk reduction of the event from medical centers, regional hospitals, district hospitals, to private clinics. The sensitivity tests revealed similar results.

**Conclusions:**

The factors associated with LFF include DM and receiving more HD sessions; on the contrary, receiving HD in private clinics is associated with less risk of LFF.

**Electronic supplementary material:**

The online version of this article (10.1186/s12882-018-1010-6) contains supplementary material, which is available to authorized users.

## Background

For patients with end-stage renal disease (ESRD) receiving hemodialysis (HD), patency of vascular access is vital for maintenance of dialysis adequacy and quality of life. Access failure might prompt patients for urgent salvage interventions, threaten their lives by dialysis inadequacy [1], and increase financial burden of the healthcare system.

Literature has confirmed that fistula is the superior modality of vascular access [2], and prevalence of fistula use was reported ranging from 49 to 92% of dialysis-dependent patients around the world [3]. However, fistula failure remains a challenge to HD patients and nephrologists, with 51% to 60% of HD patients encountering fistula failure episodes [4]. Molecular mechanisms of fistula failure are complicated and still not well understood. The most acceptable hypothesis nowadays states that once fistula is created, thrombus, uremic toxins, shear stress, hypoxemia, and inflammation will alter expression of endothelial genes and proteins, resulting in neointimal hyperplasia of fistula walls [5].

Late fistula failure (LFF), including stenosis and occlusion of fistula, is defined as failure episodes beyond 3 months after using fistula [6]. This condition contributes to the majority of failure events and is the leading cause of morbidities related to HD [7]. Numerous association factors with LFF were disclosed in the literature: Wood et al. revealed patients with old age and peripheral vascular disease (PVD) had higher risk of fistula failure [8]; Lok et al. reported male patients, patients with coronary artery disease and Caucasian ethnicity had significant fistula loss [9]. Smith et al. summarized factors affecting fistula patency, which included age, diabetes mellitus (DM), hypotension, vascular characters, smoking, ultrasound, surgical technique, and certain medicine [10]. However, the results are divergent relating to various study design and consider controversial at present.

Taiwan has become the country with the highest prevalence of ESRD [11] after implementation of the National Health Insurance (NHI) program in 1995. The ubiquitous coverage of NHI program provides integrated medical recording of registered participants. Given the divergent results of previous literature on LFF, we conducted a case-control study using the NHI Research Database (NHIRD) to identify factors affecting LFF in patients undergoing chronic HD in Taiwan.

## Methods

### Data source

We designed a population–based case–control study to investigate factors associated with LFF in patients undergoing chronic HD by means of the NHIRD. The NHI program in Taiwan was launched on 1st March 1995. NHI coverage rate totaled 99.9% according to the National Health Insurance Annual Report in 2014 [12]. All identifications in the NHIRD were encrypted to ensure privacy of patients. We used the Longitudinal Health Insurance Database 2005 (LHID2005), a subset of NHIRD, which contained complete inpatient and ambulatory care claims for a random sample of 1 million beneficiaries enrolled in the year 2005 Registry for Beneficiaries. No significant difference was observed in distribution of sex, age, and average insured payroll-related amount between LHID2005 and the original NHIRD [13]. This study complied with the Declaration of Helsinki of World Medical Association in 2000 and was approved by the Institutional Review Board of Ditmanson Medical Foundation Chia-Yi Christian Hospital in Taiwan (CYCH–IRB No.106042). The informed consent was waived because of absence of interference of decision making processes related to patient care.

### Study population

We recruited incident HD patients by using the NHI procedure codes of receiving HD from LHID2005 in 2000 and 2012. Chronic HD patients were defined as those receiving more than seven HD sessions monthly and HD regimen continued for at least 3 months. The enrollment was further restricted to those with arteriovenous fistula by using the NHI procedure codes of receiving fistula creation operation. Excluded patients comprised those aged less than 18 years, received peritoneal dialysis or kidney transplantation, or percutaneous transluminal angioplasty (PTA) before starting regular HD. Overall, the present study analyzed 1558 patients on chronic HD.

### Identification of case and control groups

**Fig. 1:**
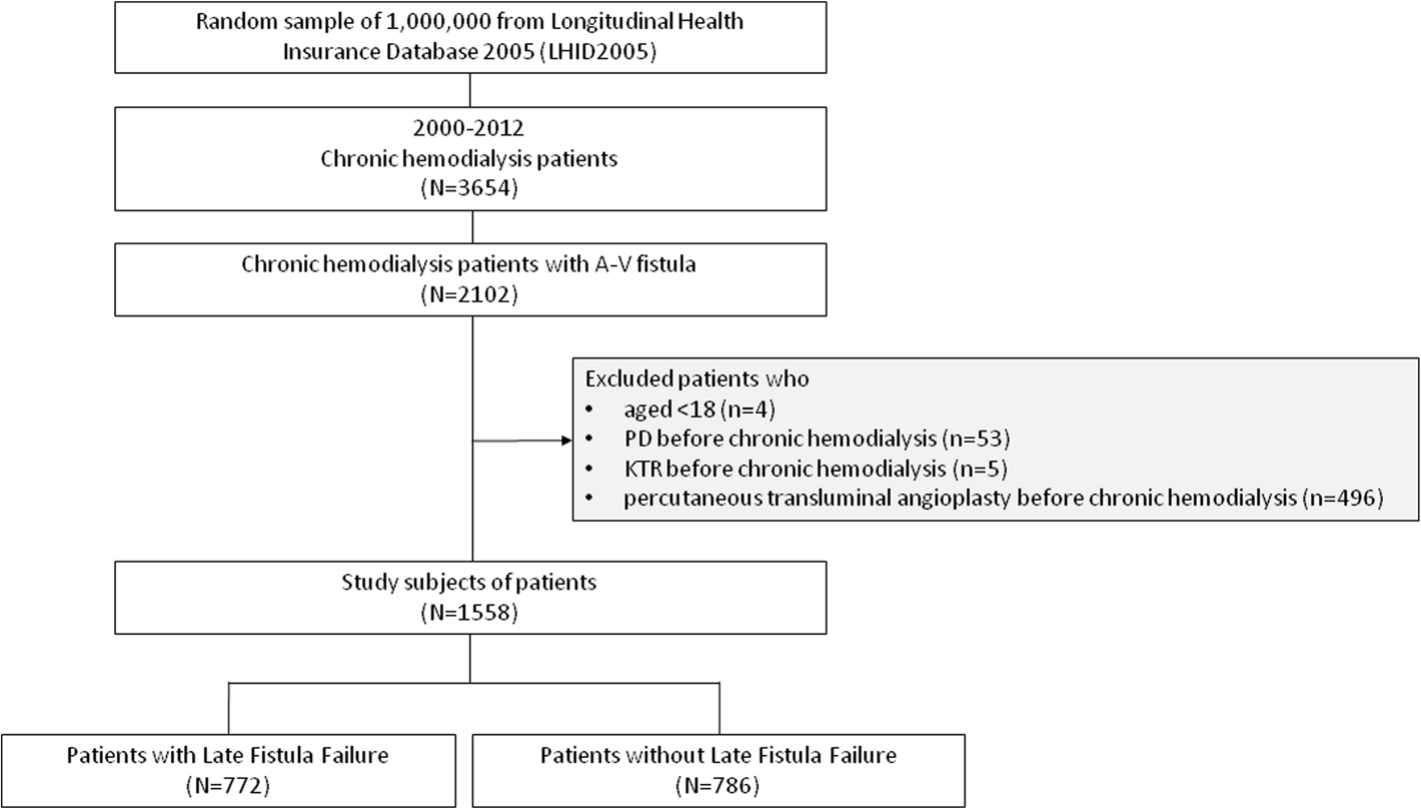
Overall flow diagram of research design and sampling strategy. A–V: arterial–venous; KTR: kidney transplant; PD: peritoneal dialysis

In our study, LFF was defined as the first fistula dysfunction episode, that required rescue treatments; beyond 3 months of chronic HD. Cases were identified as patients receiving the first episode of PTA or surgical reconstruction of permanent vascular access after a 3–month–chronic HD. PTA and surgeries were identified by using the corresponding NHI procedure codes. Controls comprised patients on chronic HD who did not receive PTA or surgical reconstructions. In total, we identified 772 cases and 786 controls in the present study, as shown in Fig. 1.

### Definitions of comorbidities, medicine and other factors

We analyzed characteristics, comorbidities, and medication of study subjects. Age was classified into four categories of 18–44, 45–64, 65–84, and over 85 years old. Income was divided into three strata according to insurance fees: low (less than 20,000 New Taiwan Dollar [NTD] per month), intermediate (between 20,000 and 40,000 NTD per month), and high (more than 40,000 NTD per month). Urbanization levels were categorized into four levels, with level 1 was defined as the most urbanized and level 4 the least urbanized community, by adjustment of a population-based stratification study [14]. Dialysis frequency was stratified into two categories: one was less than 10 sessions per month, denoting twice or less HD a week; and the other was 10 sessions or more per month, denoting thrice HD a week. Provider level was defined as the facility where patients began receiving chronic HD. Comorbidities were defined as covariates, subjects experienced at least one hospitalization or two ambulatory visits within 1 year before starting HD due to any of the following illnesses: hypertension (HTN, International Classification of Diseases, 9th Revision, Clinical Modification [ICD–9–CM] code 401–405), ischemic heart disease (IHD, ICD–9–CM code 410–414), congestive heart failure (CHF, ICD–9–CM code 402.01, 402.11, 402.91, 425, 428, and 429.3), PVD (ICD–9–CM code 440–444, and 447), arrhythmia (ICD–9–CM code 426,427, V450, and V533), cerebrovascular accident (CVA, ICD–9–CM code 430–438), DM (ICD–9–CM code 250), hyperlipidemia (ICD–9–CM code 272), hypotension (ICD–9–CM code 458), shock (ICD–9–CM code 785.5), and bloodstream–related infection (ICD–9–CM code 038, 041.9, 790.7). We also applied the Charlson comorbidity index (CCI) to denote burden of comorbidities [15]. Patients with DM were defined as DM with end organ damage [16]. We categorized CCI into three groups as scores 0–2, 3–4, and ≥ 5 according to tertiles of our data distribution. Medicine, including anticoagulants, antiplatelet agents, phosphodiesterase inhibitors, and statins, was defined as the prescription over 30 days per year within 1 year before starting HD. We also assessed midodrine, which was commonly prescribed for intradialytic hypotension, and defined it as the prescription over two times per year during HD vintage. As a sensitivity approach, we re-run the models and analyzed patients started chronic HD through their fistula without indwelling of  non-tunneled or tunneled dialysis catheters.

### Statistical analysis

Differences in patient characteristics, comorbidities, and medicine prescriptions were assessed by independent t–test and Chi-squared test. To investigate association factors of LFF in patients on chronic HD, we calculated the odds ratios (ORs) and 95% confidence intervals (CIs) by conducting logistic regression model. We also estimated the linear trend of hospital level of patients on chronic HD. All analyses were performed by operating the SPSS software for Windows (version 21.0; IBM Corporation, Somers, NY, USA). A two–tailed *p* value less than 0.05 was considered statistically significant.

## Results

As shown in Table 1, 1,558 patients were enrolled in the study. A total of 772 (49.6%) patients experienced at least once LFF episode during their HD course, whereas 786 patients were free of LFF until termination of HD or the last day of 2012. Distributions of age (62.71 ± 13.22 years vs. 62.36 ± 13.45 years) and sex (female ratio: 49.35% vs. 44.66%) were similar between the LFF group and non–LFF group. A significantly higher percentage of LFF was observed among patients receiving more than 10 HD sessions per month (78.11% vs. 67.43%, *p* < 0.001). As to provider level of chronic HD, higher percentage of LFF was observed in medical centers (17.36% vs. 12.98%), regional hospitals (30.57% vs. 27.74%) and district hospitals (19.82% vs. 19.08%), but lower percentage of LFF was observed in private clinics (32.25% vs. 40.2%). CCI of LFF group and non-LFF group were similar (3.68 ± 2.43 vs. 3.86 ± 2.53, *p* = 0.152). No difference was detected in income, comorbidities, and medicine of patients between two groups.

**Table 1:** Demographic characteristics of patients on chronic hemodialysis

	Total (*N* = 1558)	LFF (*N* = 772)	Non-LFF (*N* = 786)	*p* value
Age, years				
18–44	162 (10.40)	74 (9.59)	88 (11.20)	0.677
45–64	659 (42.30)	327 (42.36)	332 (42.24)	
65–84	708 (45.44)	358 (46.37)	350 (44.53)	
≥ 85	29 (1.86)	13 (1.68)	16 (2.04)	
Mean ± SD	62.53 ± 13.34	62.71 ± 13.22	62.36 ± 13.45	0.601
Sex				
Female	732 (46.98)	381 (49.35)	351 (44.66)	0.063
Male	826 (53.02)	391 (50.65)	435 (55.34)	
Income, NTD per month				
< 20,000	1174 (75.35)	586 (75.91)	588 (74.81)	0.485
20,000–400,000	248 (15.92)	115 (14.90)	133 (16.92)	
≥ 40,000	136 (8.73)	71 (9.20)	65 (8.27)	
Urbanization				
1	429 (27.71)	202 (26.34)	227 (29.07)	0.048
2	808 (52.20)	427 (55.67)	381 (48.78)	
3	278 (17.96)	123 (16.04)	155 (19.85)	
4	33 (2.13)	15 (1.96)	18 (2.30)	
Provider level				
Medical center	236 (15.15)	134 (17.36)	102 (12.98)	0.005
Regional hospital	454 (29.14)	236 (30.57)	218 (27.74)	
District hospital	303 (19.45)	153 (19.82)	150 (19.08)	
Private clinic	565 (36.26)	249 (32.25)	316 (40.20)	
Dialysis frequency, per month				
< 10	425 (27.28)	169 (21.89)	256 (32.57)	< 0.001
≥ 10	1133 (72.72)	603 (78.11)	530 (67.43)	
CCI				
0–2	505 (32.41)	258 (33.42)	247 (31.42)	0.415
3–4	532 (34.15)	268 (34.72)	264 (33.59)	
≥ 5	521 (33.44)	246 (31.87)	275 (34.99)	
Mean ± SD	3.77 ± 2.48	3.68 ± 2.43	3.86 ± 2.53	0.152
Comorbidity				
Hypertension	1471 (94.42)	734 (95.08)	737 (93.77)	0.260
Ischemic heart disease	701 (44.99)	343 (44.43)	358 (45.55)	0.658
Congestive heart failure	640 (41.08)	306 (39.64)	334 (42.49)	0.252
Peripheral vascular disease	268 (17.20)	134 (17.36)	134 (17.05)	0.872
Arrhythmia	304 (19.51)	145 (18.78)	159 (20.23)	0.471
Diabetes mellitus	955 (61.30)	489 (63.34)	466 (59.29)	0.100
Hyperlipidemia	897 (57.57)	446 (57.77)	451 (57.38)	0.875
Cerebrovascular accident	455 (29.20)	212 (27.46)	243 (30.92)	0.134
Hypotension	60 (3.85)	29 (3.76)	31 (3.94)	0.848
Shock	50 (3.21)	24 (3.11)	26 (3.31)	0.824
Bloodstream related infection	301 (19.32)	141 (18.26)	160 (20.36)	0.299
Drug use				
Anticoagulant	61 (3.92)	31 (4.02)	30 (3.82)	0.840
Antiplatelet Agent	794 (50.96)	384 (49.74)	410 (52.16)	0.339
Phosphodiesterase Inhibitor	930 (59.69)	468 (60.62)	462 (58.78)	0.458
Statin	788 (50.58)	401 (51.94)	387 (49.24)	0.285
Midodrine^a^	83 (5.33)	40 (5.18)	43 (5.47)	0.799

^a^Midodrine was used over 2 times per year during HD vintage

*CCI* Charlson comorbidity index, *LFF* late fistula failure, *NTD* new Taiwan dollar, *SD* standard deviation

**Fig. 2:**
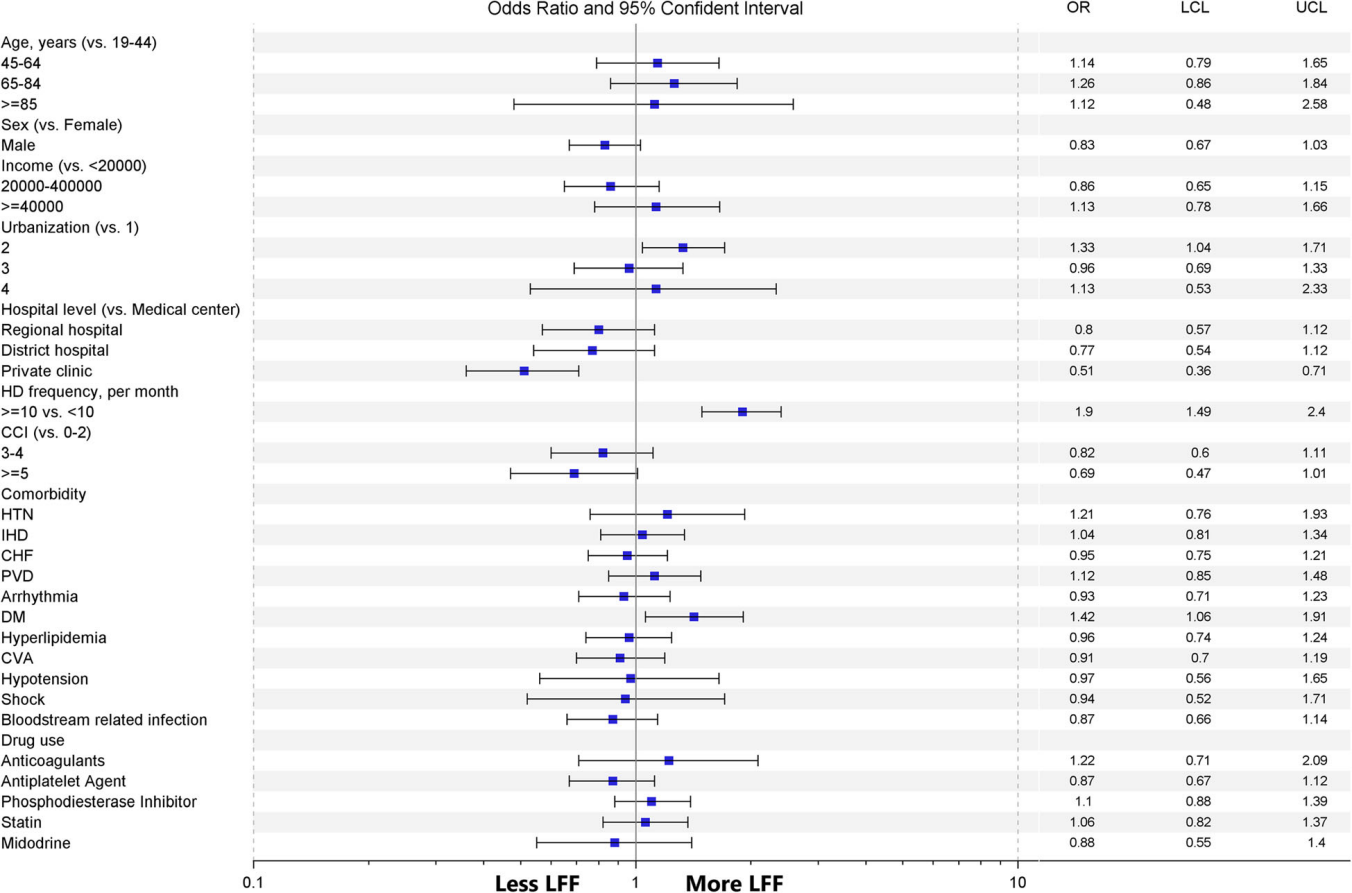
Multivariable analyses of late fistula failure of patients on chronic hemodialysis. CCI: Charlson comorbidity index; CHF: congestive heart failure; CVA: cerebrovascular accident; DM: diabetes mellitus; HTN: hypertension; IHD: ischemic heart disease; LCL: lower confidence limit; LFF: late fistula failure; NTD: new Taiwan dollar; OR: odds ratio; PVD: peripheral vascular disease UCL: upper confidence limit

To investigate the association factors of LFF in patients on chronic HD, we conducted logistic regression model, as shown in Fig. 2 (original data was shown in Additional file 1: Table S1). In multivariable analyses, there was a 49% reduced rate of LFF observed in patients in private clinics (OR: 0.51; 95% CI: 0.36–0.71) while a 90% increased rate in patients received more than 10 HD sessions per month (OR: 1.9; 95% CI: 1.49–2.40). Patients with DM presented a 42% higher rate of LFF than those without DM (OR: 1.42; 95% CI: 1.06–1.91). We did not find statistical association between LFF and other underlying characteristics, urbanization levels, CCI, and medicine.

**Fig. 3:**
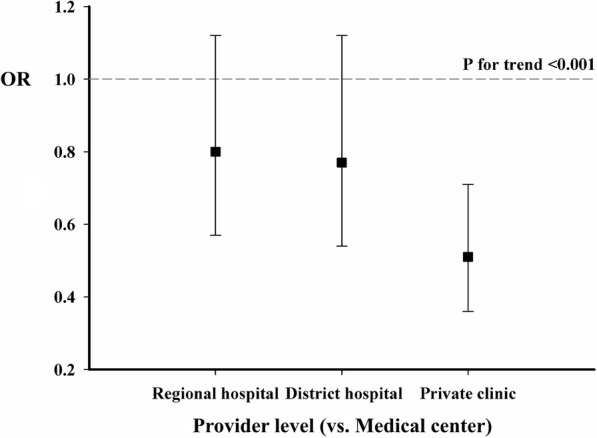
Comparison of late fistula failure among different provider levels of chronic hemodialysis. OR: odds ratio

As shown in Fig. 3, the trend test revealed a significant trend of risk reduction of LFF on provider levels of chronic HD (*p* < 0.001). We further performed stratification analysis, presented in Table 2, by means of provider level and disclosed no significant difference in age, sex, or CCI among different provider levels. However, we observed that a significant higher percentage of HD patients receiving HD thrice weekly in private clinics (83.19%) than district hospitals (68.32%), regional hospitals (66.08%) and medical centers (66.10%, *p* < 0.001). The results of sensitivity tests were similar to our main outcomes (as shown in Additional file 2: Figure S1 and Additional file 3: Table S2).

**Table 2:** Characteristics of patients categorized by dialysis provider level

	Medical center (*N* = 236)	Regional hospital (*N* = 454)	District hospital (*N* = 303)	Private clinic (*N* = 565)	*p* value
Age, years					
18–44	27 (11.44)	43 (9.47)	32 (10.56)	60 (10.62)	0.594
45–64	103 (43.64)	207 (45.59)	113 (37.29)	236 (41.77)	
65–84	100 (42.37)	198 (43.61)	152 (50.17)	258 (45.66)	
≥ 85	6 (2.54)	6 (1.32)	6 (1.98)	11 (1.95)	
Mean ± SD	61.81 ± 14.37	62.12 ± 13.00	63.29 ± 13.07	62.76 ± 13.29	0.256
Sex					
Female	106 (44.92)	215 (47.36)	137 (45.21)	274 (48.50)	0.722
Male	130 (55.08)	239 (52.64)	166 (54.79)	291 (51.50)	
Income					
< 20,000	168 (71.19)	352 (77.53)	229 (75.58)	425 (75.22)	0.383
20,000–400,000	39 (16.53)	71 (15.64)	49 (16.17)	89 (15.75)	
≥ 40,000	29 (12.29)	31 (6.83)	25 (8.25)	51 (9.03)	
Urbanization					
1	123 (52.12)	84 (18.50)	79 (26.42)	143 (25.58)	< 0.001
2	113 (47.88)	274 (60.35)	128 (42.81)	293 (52.42)	
3	0 (0.00)	96 (21.15)	77 (25.75)	105 (18.78)	
4	0 (0.00)	0 (0.00)	15 (5.02)	18 (3.22)	
Dialysis frequency, per month					
< 10	80 (33.90)	154 (33.92)	96 (31.68)	95 (16.81)	< 0.001
≥ 10	156 (66.10)	300 (66.08)	207 (68.32)	470 (83.19)	
CCI					
0–2	87 (36.86)	138 (30.40)	85 (28.05)	195 (34.51)	0.220
3–4	74 (31.36)	157 (34.58)	105 (34.65)	196 (34.69)	
≥ 5	75 (31.78)	159 (35.02)	113 (37.29)	174 (30.80)	
Mean ± SD	3.66 ± 2.59	3.81 ± 2.39	4.01 ± 2.62	3.64 ± 2.42	0.723
Comorbidity					
Hypertension	217 (91.95)	434 (95.59)	290 (95.71)	530 (93.81)	0.152
Ischemic heart disease	100 (42.37)	193 (42.51)	155 (51.16)	253 (44.78)	0.092
Congestive heart failure	84 (35.59)	201 (44.27)	152 (50.17)	203 (35.93)	< 0.001
Peripheral vascular disease	31 (13.14)	58 (12.78)	59 (19.47)	120 (21.24)	0.001
Arrhythmia	52 (22.03)	86 (18.94)	74 (24.42)	92 (16.28)	0.024
Diabetes mellitus	145 (61.44)	283 (62.33)	187 (61.72)	340 (60.18)	0.912
Hyperlipidemia	127 (53.81)	259 (57.05)	188 (62.05)	323 (57.17)	0.268
Cerebrovascular disease	54 (22.88)	139 (30.62)	106 (34.98)	156 (27.61)	0.014
Hypotension	9 (3.81)	20 (4.41)	14 (4.62)	17 (3.01)	0.584
Shock	5 (2.12)	17 (3.74)	15 (4.95)	13 (2.30)	0.123
Bloodstream related infection	44 (18.64)	103 (22.69)	57 (18.81)	97 (17.17)	0.165
Drug use					
Anticoagulant	6 (2.54)	17 (3.74)	18 (5.94)	20 (3.54)	0.192
Antiplatelet Agent	116 (49.15)	228 (50.22)	177 (58.42)	273 (48.32)	0.033
Phosphodiesterase Inhibitor	117 (49.58)	288 (63.44)	196 (64.69)	329 (58.23)	0.001
Statin	113 (47.88)	235 (51.76)	159 (52.48)	281 (49.73)	0.673
Midodrine^a^	20 (8.47)	29 (6.39)	14 (4.62)	20 (3.54)	0.023

^a^Midodrine was used over 2 times per year during HD vintage

*CCI* Charlson comorbidity index, *NTD* new Taiwan dollar, *SD* standard deviation

## Discussion

In the present study, we observed that DM, dialysis frequency, and provider level of chronic HD affected risk of LFF. Diabetic patients showed a 42% increased rate of LFF. Patients who received more than 10 HD sessions per month featured a 90% increased rate of LFF. HD patients receiving regular HD at private clinics exhibited a 49% reduced rate of LFF than those who received HD in medical centers. A significant risk reduction of LFF was observed from medical centers, regional hospitals, district hospitals, and private clinics.

Endothelial dysfunction and increased thrombogenicity related to hyperglycemia affect patency of fistula and contribute DM as a risk factor of LFF [17]. Our results showed that LFF was more prevalent in diabetic patients. This result agreed with the findings from transnational studies [11, 18, 19]; though other study discovered that DM exerted no adverse effect on fistula complications [20]. The controversy might result from the difference of study subjects in terms of distribution of age and sex. Further investigation is warranted in consideration of vintage and severity (example: level of glycated hemoglobin) of DM for its divergent results on LFF.

Receiving over 10 HD sessions per month denotes administrating thrice HD sessions per week. In our study, nearly three-fourths of patients received HD sessions thrice weekly, and they featured a 90% higher LFF rates than those who received HD sessions twice weekly. We made an internal validation by analyzing those without dialysis catheter indwelling before LFF. The effect of dialysis frequency showed more significant (OR: 2.3, 95% CI: 1.53–3.45) in subgroup analysis (Additional file 2: Figure S1). Dialysis frequency played a crucial role of dialysis adequacy, which influenced quality of life and mortality [21], and did not determine by the preference of nephrologists and patients. We observed that dialysis frequency might affect LFF due to the numbers of fistula usage. Increased dialysis frequency results in more significant risk of puncture mistake and hemostasis failure, which might cause LFF. Our finding was consistent with the research of Suri et al., which reported frequent HD raised the risk of vascular access complications [22].

As to the association between LFF and dialysis provider level, we observed that rate of LFF was significantly lower in private clinics in contrast to medical centers, regional hospitals, and district hospitals. Subgroup analysis of those without dialysis catheter indwelling was operated and exhibited similar results (OR: 0.57, 95% CI: 0.32–1.00, Additional file 2: Figure S1). Asano et al. compared treatment practices of dialysis facility from the Dialysis Outcomes and Practice Patterns Study (DOPPS) and concluded no significant association between fistula survival and physician and staff practices [23]. It might be limited since practices of physician and staff were defined by brief questionnaires and unquantifiable factors including education and nursing practice on fistula care, were not evaluated. We observed a 49% risk reduction of LFF in patients who received HD in private clinics after multivariable adjustment models (Fig. 2). In our study, patients in private clinics presented significantly higher ratio of receiving HD thrice weekly, with dialysis frequency analyzed as a factor of LFF. The burden of comorbidities, presented by CCI, was observed similar among different dialysis provider levels after validations (Table 2 and Additional file 3: Table S2). Taiwan Society of Nephrology has clearly regulated staffing of HD unit [24], though workload of HD facility was observed varied in different provider levels. We supposed that HD staff in private clinics might pay full attention to patient care, including fistula care and patient education, for example, rather than administrative loading. It might be a plausible cause of higher percentage of thrice–a–week in dialysis patients yet lower risk of LFF in private clinics.

Fistula care includes postoperative rehabilitation, physical examination, predialytic skin preparation, cannulation, intradialytic settings, critical management, hemostasis, and postdialytic surveillance. Guidelines and large–scale studies had confirmed that good quality of fistula care positively influenced survival of vascular access [25–28]. With regard to the association between LFF and fistula care factors, cannulation is a well-established factor. [29–31]. Additionally, literature ever reported that nursing staff affected LFF by their technique and education [24, 27, 32]. However, most factors outlined above were lack of objective definitions and quantifiable variables for investigation. Effect of dialysis frequency and dialysis provider level on LFF might imply direct and indirect evidence of fistula care, respectively.

We accessed the association between LFF and characteristics, comorbidities, and medicine of patients in the NHIRD. Our results showed that age was not a significant factor of LFF, agreeing with previous studies [19, 33]. Lazarides et al. observed that increased age might deteriorate LFF rate in a meta-analysis research; though only radiocephalic fistula was included [34]. We did not separate fistula location since radiocephalic fistula was not always the optimum choice for vascular access; thus our results might be more generalized to real world practice status. Our findings also showed no significant influence of sex on LFF, and were compatible with those of previous studies, including a meta-analysis article [33, 35].

As to comorbidities, PVD is another frequently-referred factor of LFF in addition to DM. Our results showed that HD patients with PVD featured a marginal risk excess of LFF than those without PVD (OR: 1.12, 95% CI: 0.85–1.48, Fig. 2), and this finding differed from those of previous studies [36, 37]. Clinical presentations of PVD varied from absence of symptoms to critical ischemia. Our definition of PVD, which was based solely on the ICD–9–CM coding, might cause underestimation of diagnosis. Further validation tests are warranted for precise and inclusive definition of PVD.

We analyzed the medicine affecting vasculature, including anticoagulants, antiplatelet agents, phosphodiesterase inhibitors, and statins, in the NHIRD. Heparin was excluded owing to its widespread use during dialysis. Midodrine was included because of its common use for intradialytic hypotension and might represent occurrence of intradialytic hypotension, a remarkable risk factor of LFF [10]. Our results revealed none of them exerted significant influence on LFF, coinciding with the findings in DOPPS and other NHIRD studies [38, 39]. Cochrane systemic review by Tanner and Da Silva have revealed that ticlopidine might beneficially affect fistula patency [40]. Our study did not show similar results in subgroup analysis, and it might be related to small sample size of ticlopidine. In addition, Chang et al. have observed that statins might improve patency of fistula [41]. Our study did not show similar results, and it might be in relation to absence of medicine wash-out setting in our study design. Our study also failed to determine the association between LFF and midodrine, and it might refer to small sample number by our definition. Studies addressing the relationship between LFF and medicine as primary outcomes are required in the future.

We conducted a population–based study with 13–year–follow–up. The NHIRD allowed for obtaining data from the whole population in Taiwan related to its universal coverage regardless of socioeconomical status and physical condition. Characteristics of the NHIRD might reflect actual medical conditions in Taiwan. As shown in Fig. 1, we defined 3654 persons as chronic HD patients from the LHID2005, and it was comparable with epidemiological results of 2016 Annual Report on Kidney Disease in Taiwan published by the National Health Research Institutes [42]. In our study, rate of LFF reached 49.6%, which agreed with results of international multicenter studies [18, 43]. We strengthened definitions of comorbidities with ICD–9–CM codes for at least one hospitalization or two ambulatory visits to modify potential information bias in the NHIRD.

Our study faced several limitations. First, LFF was examined by clinical symptoms and image findings. However, those abnormalities were not available in forms of parameter in the NHIRD. Second, preoperative mapping, fistula location, vascular characters, surgical technique, first needling time, cannulation method, far infrared therapy, body mass index, and cigarette use were referred to important factors leading to LFF [10, 31] but lacking in our database. Third, our results might suppose the importance of fistula care and quality of care among different dialysis providers, but no proper surrogate has been developed for fistula care by far. Fourth, exposure to medicine was based on prescription information only. We could not determine whether patients adhered to prescribed schedule, and this condition might result in misclassification of exposure and underestimation of findings. Lastly, biomarkers such as calcium, phosphate, parathyroid hormone, C-reactive protein, and lipoproteins, were associated with LFF in previous studies [44, 45] but not available in the NHIRD. Integrated trials including laboratory data of study subjects should be conducted in the future to elucidate our results.

## Conclusions

DM, dialysis frequency, and provider levels of chronic HD affected the risk of LFF. Diabetic patients featured a 42% increased rate of LFF. Patients with dialysis frequency of more than 10 HD sessions per month presented a 90% increased rate of LFF. Patients receiving chronic HD in private clinics showed a 49% reduction rate of LFF. A significant trend of risk reduction of LFF was observed from medical centers, regional hospitals, district hospitals, to private clinics. Dialysis frequency and provider level of chronic HD might imply linkage of LFF and fistula care. Quality of fistula care might be emphasized to reduce risk of LFF.

## Additional files

Additional file 1: **Table S1.** Analysis of factors affecting late fistula failure in patients on chronic hemodialysis. (DOCX 17 kb)

Additional file 2: **Figure S1.** Multivariable analyses of late fistula failure of patients on chronic hemodialysis without dialysis catheter indwelling. CCI: Charlson comorbidity index; CHF: congestive heart failure; CVA: cerebrovascular accident; DM: diabetes mellitus; HTN: hypertension; IHD: ischemic heart disease; LCL: lower confidence limit; LFF: late fistula failure; NTD: new Taiwan dollar; OR: odds ratio; PVD: peripheral vascular disease UCL: upper confidence limit. (TIF 1081 kb)

Additional file 3: **Table S2.** Characteristics of patients categorized by dialysis provider level without dialysis catheter indwelling. (DOCX 18 kb)

## Abbreviations

CCI: Charlson comorbidity index
CHF: Congestive heart failure
CI: Confidence interval
CVA: cerebrovascular accident
DM: Diabetes mellitus
DOPPS: Dialysis Outcomes and Practice Patterns Study
ESRD: End-stage renal disease
HD: Hemodialysis
HTN: Hypertension
ICD–9–CM: International Classification of Diseases, 9th Revision, Clinical Modification
IHD: Ischemic heart disease
LFF: Late fistula failure
LHID: Longitudinal Health Insurance Database
NHI: National Health Insurance
NHIRD: National Health Insurance Research Database
NTD: New Taiwan Dollar
OR: Odds ratio
PTA: Percutaneous transluminal angioplasty
PVD: Peripheral vascular disease
