# Supplementary material for: Factors affecting fistula failure in patients on chronic hemodialysis: a population–based case–control study
Source: BMC Nephrol. 2018 Aug 22;19:213. doi: 10.1186/s12882-018-1010-6 (PMC6106750; doi:10.1186/s12882-018-1010-6)
Supplement: Supplementary file 1 — Table S1. Analysis of factors affecting late fistula failure in patients on chronic hemodialysis. (DOCX 17 kb) [file 12882_2018_1010_MOESM1_ESM.docx]

**Table S1.** Analysis of factors affecting late fistula failure in patients on chronic hemodialysis

|  | Univariable analysis | | | Multivariable analysis | |
| --- | --- | --- | --- | --- | --- |
|  | OR | 95%CI | OR | | 95%CI |
| Age, years (vs. 19-44) |  |  |  | |  |
| 45-64 | 1.17 | 0.83-1.66 | 1.14 | | 0.79-1.65 |
| 65-84 | 1.22 | 0.87-1.73 | 1.26 | | 0.86-1.84 |
| ≥85 | 0.97 | 0.44-2.15 | 1.12 | | 0.48-2.58 |
| Sex (vs. Female) |  |  |  | |  |
| Male | 0.82 | 0.67-1.01 | 0.83 | | 0.67-1.03 |
| Income, NTD per month (vs. <20000) |  |  |  | |  |
| 20000-400000 | 0.87 | 0.66-1.14 | 0.86 | | 0.65-1.15 |
| ≥40000 | 1.10 | 0.77-1.56 | 1.13 | | 0.78-1.66 |
| Urbanization (vs. 1) |  |  |  | |  |
| 2 | 1.26 | 0.99-1.59 | 1.33 | | 1.04-1.71 |
| 3 | 0.89 | 0.66-1.21 | 0.96 | | 0.69-1.33 |
| 4 | 0.94 | 0.46-1.91 | 1.13 | | 0.53-2.33 |
| Provider level (vs. Medical center) |  |  |  | |  |
| Regional hospital | 0.82 | 0.60-1.13 | 0.80 | | 0.57-1.12 |
| District hospital | 0.77 | 0.54-1.08 | 0.77 | | 0.54-1.12 |
| Private clinic | 0.60 | 0.44-0.82 | 0.51 | | 0.36-0.71 |
| Dialysis frequency, per month |  |  |  | |  |
| ≥10 vs. <10 | 1.74 | 1.39-2.19 | 1.90 | | 1.49-2.40 |
| CCI (vs. 0-2) |  |  |  | |  |
| 3-4 | 0.98 | 0.76-1.25 | 0.82 | | 0.60-1.11 |
| ≥5 | 0.85 | 0.67-1.09 | 0.69 | | 0.47-1.01 |
| Comorbidity |  |  |  | |  |
| Hypertension | 1.28 | 0.83-1.99 | 1.21 | | 0.76-1.93 |
| Ischemic heart disease | 0.96 | 0.78-1.17 | 1.04 | | 0.81-1.34 |
| Congestion heart failure | 0.89 | 0.73-1.09 | 0.95 | | 0.75-1.21 |
| Peripheral vascular disease | 1.02 | 0.79-1.33 | 1.12 | | 0.85-1.48 |
| Arrhythmia | 0.90 | 0.70-1.16 | 0.93 | | 0.71-1.23 |
| Diabetes mellitus | 1.18 | 0.96-1.45 | 1.42 | | 1.06-1.91 |
| Hyperlipidemia | 1.01 | 0.83-1.24 | 0.96 | | 0.74-1.24 |
| Cerebrovascular accident | 0.84 | 0.68-1.05 | 0.91 | | 0.70-1.19 |
| Hypotension | 0.95 | 0.57-1.59 | 0.97 | | 0.56-1.65 |
| Shock | 0.90 | 0.51-1.59 | 0.94 | | 0.52-1.71 |
| Bloodstream related infection | 0.88 | 0.67-1.12 | 0.87 | | 0.66-1.14 |
| Drug use |  |  |  | |  |
| Anticoagulants | 1.02 | 0.61-1.71 | 1.22 | | 0.71-2.09 |
| Antiplatelet Agent | 0.90 | 0.74-1.10 | 0.87 | | 0.67-1.12 |
| Phosphodiesterase Inhibitor | 1.10 | 0.90-1.35 | 1.10 | | 0.88-1.39 |
| Statin | 1.12 | 0.91-1.36 | 1.06 | | 0.82-1.37 |
| Midodrine | 0.94 | 0.61-1.47 | 0.88 | | 0.55-1.40 |

**Midodrine was used over 2 times per year during HD vintage

CCI: Charlson comorbidity index; CI: confident interval; NTD: new Taiwan dollar; OR: odds ratio
