# Supplementary figures and images for: Factors affecting fistula failure in patients on chronic hemodialysis: a population–based case–control study
Source: BMC Nephrol. 2018 Aug 22;19:213. doi: 10.1186/s12882-018-1010-6 (PMC6106750; doi:10.1186/s12882-018-1010-6)

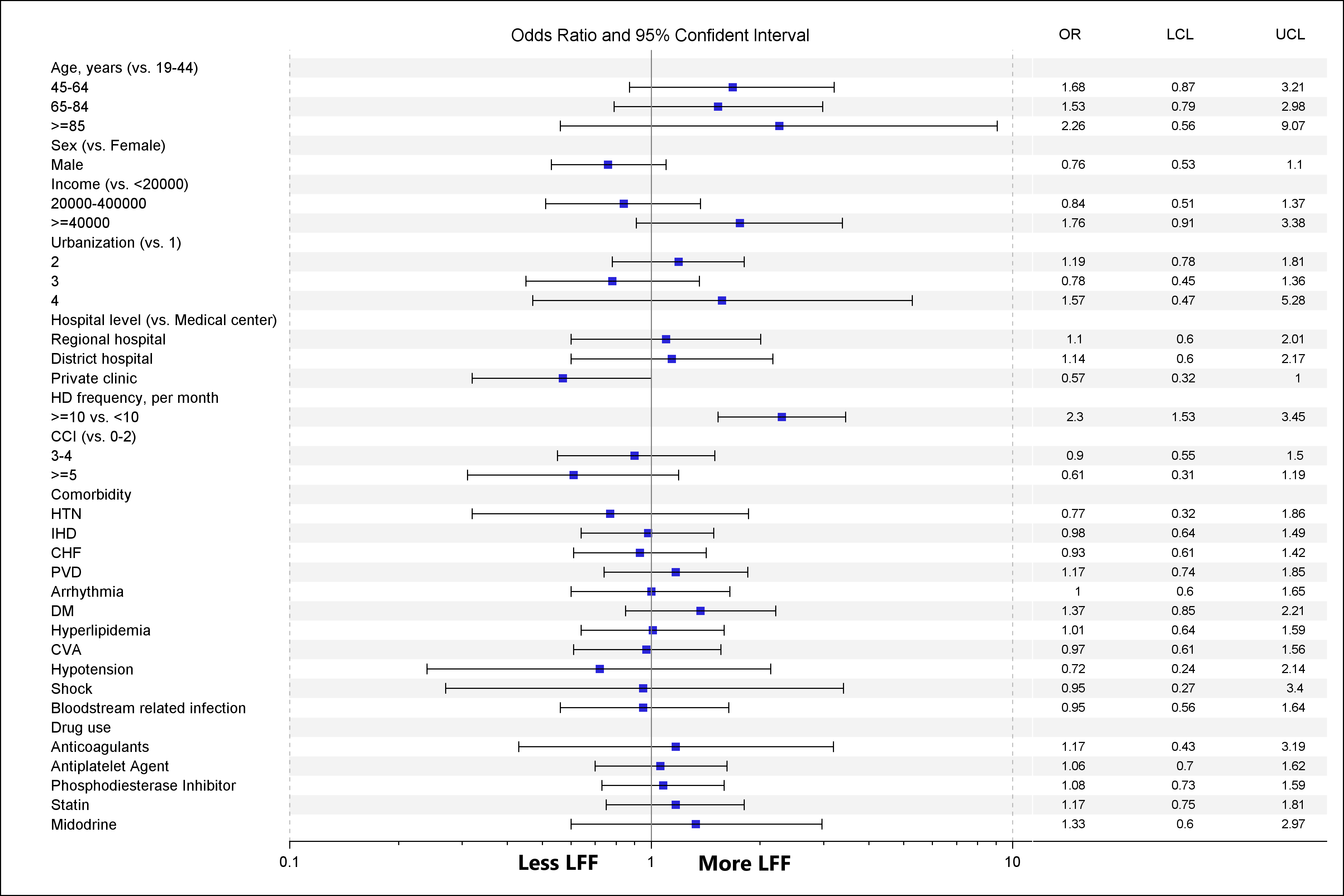

Supplement: Supplementary file 2 — Figure S1. Multivariable analyses of late fistula failure of patients on chronic hemodialysis without dialysis catheter indwelling. CCI: Charlson comorbidity index; CHF: congestive heart failure; CVA: cerebrovascular accident; DM: diabetes mellitus; HTN: hypertension; IHD: ischemic heart disease; LCL: lower confidence limit; LFF: late fistula failure; NTD: new Taiwan dollar; OR: odds ratio; PVD: peripheral vascular disease UCL: upper confidence limit. (TIF 1081 kb) [file 12882_2018_1010_MOESM2_ESM.tif]
