# Supplementary material for: Factors affecting fistula failure in patients on chronic hemodialysis: a population–based case–control study
Source: BMC Nephrol. 2018 Aug 22;19:213. doi: 10.1186/s12882-018-1010-6 (PMC6106750; doi:10.1186/s12882-018-1010-6)
Supplement: Supplementary file 3 — Table S2. Characteristics of patients categorized by dialysis provider level without dialysis catheter indwelling. (DOCX 18 kb) [file 12882_2018_1010_MOESM3_ESM.docx]

**Table S2** Characteristics of patients categorized by provider level without dialysis catheter indwelling

|  | Medical center (N=79) | Regional hospital (N=158) | District hospital (N=109) | Private clinic (N=216) | *p* value |
| --- | --- | --- | --- | --- | --- |
| Age, years |  |  |  |  |  |
| 19-44 | 4 (5.06) | 18 (11.39) | 10 (9.17) | 25 (11.57) | 0.421 |
| 45-64 | 38 (48.10) | 80 (50.63) | 49 (44.95) | 84 (38.89) |  |
| 65-84 | 35 (44.30) | 58 (36.71) | 48 (44.04) | 101 (46.76) |  |
| ≥85 | 2 (2.53) | 2 (1.27) | 2 (1.83) | 6 (2.78) |  |
| Mean±SD | 62.74±12.12 | 61.21±12.52 | 62.24±12.48 | 63.38±13.65 | 0.294 |
| Sex |  |  |  |  |  |
| Female | 39 (49.37) | 73 (46.20) | 51 (46.79) | 116 (53.70) | 0.47 |
| Male | 40 (50.63) | 85 (53.80) | 58 (53.21) | 100 (46.30) |  |
| Income, NTD per month |  |  |  |  |  |
| <20000 | 59 (74.68) | 125 (79.11) | 76 (69.72) | 157 (72.69) | 0.295 |
| 20000-400000 | 10 (12.66) | 22 (13.92) | 25 (22.94) | 39 (18.06) |  |
| ≥40000 | 10 (12.66) | 11 (6.96) | 8 (7.34) | 20 (9.26) |  |
| Urbanization |  |  |  |  |  |
| 1 | 44 (55.70) | 25 (15.82) | 30 (27.52) | 62 (29.11) | <0.001 |
| 2 | 35 (44.30) | 97 (61.39) | 47 (43.12) | 106 (49.77) |  |
| 3 | 0 (0.00) | 36 (22.78) | 28 (25.69) | 36 (16.90) |  |
| 4 | 0 (0.00) | 0 (0.00) | 4 (3.67) | 9 (4.23) |  |
| HD frequency, per month |  |  |  |  |  |
| <10 | 25 (31.65) | 50 (31.65) | 39 (35.78) | 43 (19.91) | 0.008 |
| ≥10 | 54 (68.35) | 108 (68.35) | 70 (64.22) | 173 (80.09 |  |
| CCI |  |  |  |  |  |
| 0-2 | 33 (41.77) | 63 (39.87) | 34 (31.19) | 85 (39.35) | 0.696 |
| 3-4 | 25 (31.65) | 51 (32.28) | 37 (33.94) | 73 (33.80) |  |
| ≥5 | 21 (26.58) | 44 (27.85) | 38 (34.86) | 58 (26.85) |  |
| Mean±SD | 3.28±2.46 | 3.44±2.39 | 3.72±2.46 | 3.39±2.24 | 0.797 |
| Comorbidity |  |  |  |  |  |
| Hypertension | 76 (96.20) | 151 (95.57) | 103 (94.50) | 207 (95.83) | 0.939 |
| Ischemic heart disease | 36 (45.57) | 61 (38.61) | 50 (45.87) | 94 (43.52) | 0.605 |
| Congestive heart failure | 25 (31.65) | 70 (44.30) | 51 (46.79) | 75 (34.72) | 0.045 |
| Peripheral vascular disease | 15 (18.99) | 21 (13.29) | 23 (21.10) | 48 (22.22) | 0.164 |
| Arrhythmia | 12 (15.19) | 21 (13.29) | 22 (20.18) | 38 (17.59) | 0.469 |
| Diabetes mellitus | 45 (56.96) | 88 (55.70) | 59 (54.13) | 121 (56.02) | 0.982 |
| Hyperlipidemia | 41 (51.90) | 96 (60.76) | 72 (66.06) | 134 (62.04) | 0.259 |
| Cerebrovascular disease | 13 (16.46) | 44 (27.85) | 36 (33.03) | 60 (27.78) | 0.088 |
| Hypotension | 5 (6.33) | 4 (2.53) | 3 (2.75) | 4 (1.85) | 0.240 |
| Shock | 1 (1.27) | 2 (1.27) | 4 (3.67) | 4 (1.85) | 0.571 |
| Bloodstream related infection | 6 (7.59) | 32 (20.25) | 13 (11.93) | 21 (9.72) | 0.012 |
| Drug use |  |  |  |  |  |
| Anticoagulants | 2 (2.53) | 3 (1.90) | 6 (5.50) | 7 (3.24) | 0.467 |
| Antiplatelet Agent | 37 (46.84) | 77 (48.73) | 57 (52.29) | 99 (45.83) | 0.732 |
| Phosphodiesterase Inhibitor | 44 (55.70) | 93 (58.86) | 68 (62.39) | 133 (61.57) | 0.759 |
| Statin | 41 (51.90) | 89 (56.33) | 58 (53.21) | 114 (52.78) | 0.890 |
| Midodrine** | 9 (11.39) | 12 (7.59) | 5 (4.59) | 5 (2.31) | 0.010 |

**Midodrine was used over 2 times per year during HD vintage

CCI: Charlson comorbidity index; NTD: new Taiwan dollar; SD: standard deviation.
